# Enhancing Surface Temperature Uniformity in a Liquid Silicone Rubber Injection Mold with Conformal Heating Channels

**DOI:** 10.3390/ma16175739

**Published:** 2023-08-22

**Authors:** Chil-Chyuan Kuo, Qing-Zhou Tasi, Song-Hua Huang, Shih-Feng Tseng

**Affiliations:** 1 Department of Mechanical Engineering, Ming Chi University of Technology, New Taipei City 24301, Taiwan; 2 Research Center for Intelligent Medical Devices, Ming Chi University of Technology, No. 84, Gungjuan Road, New Taipei City 24301, Taiwan; 3 Department of Mechanical Engineering, Chang Gung University, No. 259, Wenhua 1st Road, Guishan District, Taoyuan City 33302, Taiwan; 4 Center for Reliability Engineering, Ming Chi University of Technology, No. 84, Gungjuan Road, New Taipei City 24301, Taiwan; 5 Li-Yin Technology Co., Ltd., No. 37, Lane 151, Section 1, Zhongxing Road, Wugu District, New Taipei City 24101, Taiwan; 6 Department of Mechanical Engineering, National Taipei University of Technology, No. 1, Section 3, Zhongxiao E. Road, Da’an District, Taipei City 106344, Taiwan

**Keywords:** liquid silicone rubber, conformal heating channel, simulation, sensor modes, average temperature

## Abstract

To enhance the productivity and quality of optical-grade liquid silicone rubber (LSR) and an optical convex lens simultaneously, uniform vulcanization of the molding material is required. However, little has been reported on the uniform vulcanization of LSR in the heated cavity. This paper presents a conformal heating channel to enhance the temperature uniformity of the mold surface in the LSR injection molding. The curing rate of an optical convex lens was numerically investigated using Moldex3D molding simulation software. Two different sets of soft tooling inserts, injection mold inserts with conventional and conformal heating channels, were fabricated to validate the simulation results. The mold surface temperature uniformity was investigated by both numerical simulation and experiment. In particular, both a thermal camera and thermocouples were employed to measure the mold surface temperature after LSR injecting molding. It was found that the uniformity of the mold surface for LSR injection mold with the conformal heating channel was better. The average temperature of the mold surface could be predicted by the heating oil temperature according to the proposed prediction equation. The experimental results showed that the trend of the average temperature of five sensor modes was consistent with the simulation results. The error rate of the simulation results was about 8.31% based on the experimental result for the LSR injection mold with the conformal heating channel.

## 1. Introduction

Liquid silicone rubber (LSR) is a two-component system with the distinctive characteristics of high-temperature resistance, electrical insulation, flame resistance, chemical stability, and corrosion resistance. Thus, the demand for LSR is experiencing exponential growth since LSR injection molding (IM) is widely used for fabricating products in many industries, such as consumer goods, food, and automotive [1,2]. Typically, silicone rubber belongs to the family of thermoset elastomers. Therefore, LSR parts are fire retardant and can withstand extreme temperatures [3]. In addition, LSR parts can resist acids and alkalis. LSR is especially suitable for high-quality production of parts that include sealing membranes, seals and electric connectors.

Rey et al. [4] provided a new method for studying the nickel–titanium–silicone structure for biomechanical applications. Results showed that the contraction length is less than the diameter expansion by about 7%, which is a main advantage of a nickel–titanium–silicone rubber composite. Ou et al. [5] studied the filling and curing phases of injection molding of liquid silicone rubbers and proposed a model using molding simulation software. The rheokinetic properties are described for different silicone fluid samples from 25 to 100 °C. Zhang et al. [6] investigated the effects of vinyl-functionalized silica particles on the mechanical and thermal properties of an LSR nanocomposite. The results revealed that vinyl-modified SiO_2_ particle/liquid silicone rubber composites had higher thermal stability and mechanical strength than non-modified composites, which gave rise to ideas about preparing a high-performance LSR with low viscosity. Magaña et al. [7] designed functionalizing silicone rubber films by a direct grafting method using gamma irradiation to induce polymerization. The results revealed that the polymeric prodrug in medical devices for breast reconstruction and augmentation can decrease the 25-day period before returning to normal. Guo et al. [8] investigated the anticondensation characteristics of LSR temperature-control coatings. It was found that the coatings containing phase-change capsules significantly enhanced the anticondensation performance of metal cabinets. Wu et al. [9] developed epoxy and boron-modified polydimethylsiloxane to provide a two-component addition-cured LSR with good self-adhesion. The results revealed that the LSR had a thermal conductivity of 1.59 W/m-K. Marl et al. [10] analyzed the cell structure of these LSR foams and found it to be very homogeneous. Woitschach et al. [11] modified the LSR with two of the most commonly used thermoplastic polyurethanes and evaluated the inflammatory potential of the materials. Shang et al. [12] used LSR filled with SiC nanoparticles to insulate cable accessories. It was found that the sample with 3 wt. % SiC/LSR had the best nonlinear conductivity and nonlinear conductivity coefficient. Kaitainen et al. [13] found that micropatterning or silver coating improved electrode performance significantly based on the electrically conductive LSR. Seitz et al. [14] used surface treatment technology to improve LSR adhesion strength, which resulted in stronger bonding to the organofunctional silane of the self-adhesive silicone rubber. Liu et al. [15] showed a hydantoin-containing silane to improve tracking and bacteria resistance. The results showed that the thermal stability of addition-cured LSR improved significantly. Harkousa et al. [16] investigated the rheological behavior of an LSR and found crosslinking kinetics. Qiu et al. [17] investigated the effects of functional silane on LSR flame retardant and thermal stability. However, very few studies have focused on the uniform vulcanization of optical-grade LSR.

In general, LSR resists extreme temperatures with good ability and stability, and injection molding manufactures durable and pliable components in high volume. It should be noted that uniform vulcanization of material in the heated cavity is required to enhance the productivity and quality of the molded parts simultaneously. The main objective is to enhance surface temperature uniformity in the LSR injection mold by using conformal heating channels. First, the mold surface temperature was numerically examined using Moldex3D molding simulation software (R14 SP3OR, CoreTech System Inc., New Taipei City, Taiwan). Then, two LSR injection molds were fabricated: one with a conformal heating channel fabricated using rapid tooling technology and one with a conventional heating channel. Finally, a comprehensive analysis of the mold surface temperature was performed by thermal camera and thermocouple. Temperature uniformity during the vacuolization of an optical convex lens was investigated by both numerical simulation and experiment by both thermal camera and thermocouple.

## 2. Experimental Details

**Figure 1:**
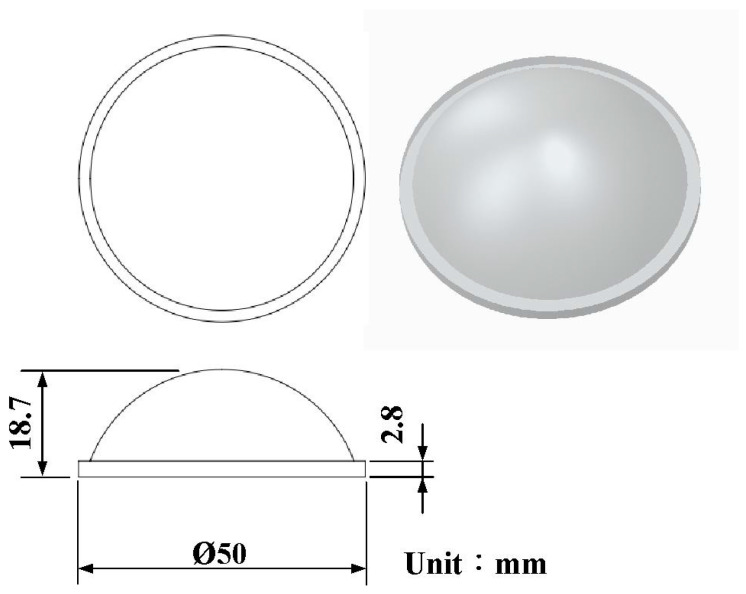
CAD model of an optical convex lens.

**Figure 2:**
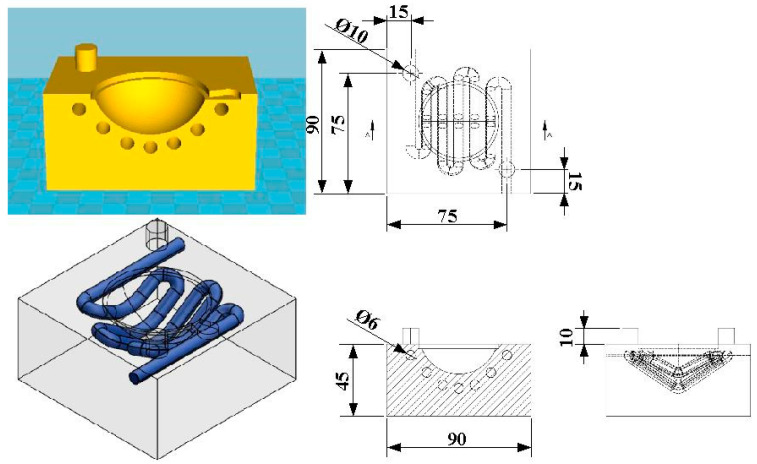
Design of the rapid tool with a conformal heating channel.

In this study, an optical convex lens was selected as the part to be molded. The part, rapid tool, conformal heating channel, and conventional heating channel were designed by 3D modeling software (Parametric Technology Inc., Boston, MA, USA). Figure 1 shows the computer-aided design (CAD) model of an optical convex lens. The diameter was 50 mm with a thickness in the center of approximately 18.7 mm. Figure 2 shows the design of the rapid tool with the conformal heating channel. The cavity insert had a length and width of 90 mm and a height of 45 mm. The core insert had a length and width of 90 mm and a height of 30 mm. The cross-section of the conformal heating channel was circular with a diameter of 6 mm.

Two different kinds of rapid tools for fabricating optical convex lens were designed and implemented with the developed mixture. One was the LSR injection mold with the conformal heating channel. The other was the mold with the conventional heating channel for comparison. The rapid tools were fabricated with the mixture composed of epoxy resins (EP-2N1, Ruixin Inc., New Taipei City, Taiwan) and 41 vol.% aluminum powder with average particle size of about 45 µm. First, an intermediate mold was fabricated using silicone rubber (KE-1310ST, Shin Etsu Inc., New Taipei City, Taiwan). To eliminate the air bubbles, the mixture was put into a vacuum pump (F-600, Feiling Inc. New Taipei City, Taiwan). Both the conventional and conformal heating channels were fabricated with polyvinyl butyral (PVB) filament feedstock (Thunder 3D Inc., New Taipei City, Taiwan) [18,19] using fused filament modeling (FFF) technology [20,21] (Teklink smart solution Inc., New Taipei City, Taiwan). The process parameters of FFF included a printing speed of 30 mm/s, bed temperature of 60 °C, printing temperature of 200 °C, and layer thickness of 0.1 mm. The molding material was ELASTOSIL^®^ LR 3003/50 US A/B (Wacker Chemical AG Inc., Munich, Germany) [22] because it is suitable for manufacturing an optical convex lens through LSR injection molding.

**Figure 3:**
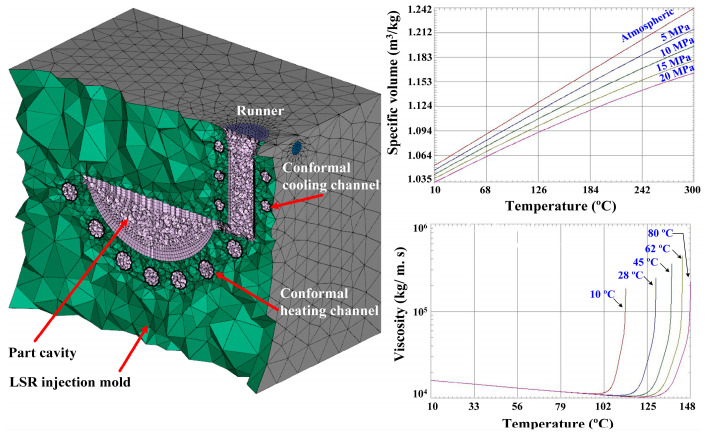
Simulation models and material properties of the molding materials.

The primary purpose of numerical simulation was to understand the temperature uniformity of conventional heating channels in the LSR molds. In this study, the Moldex3D simulation software (R14 SP3OR, CoreTech System Inc., New Taipei City, Taiwan) was used to investigate the curing rate of a fisheye lens. Figure 3 shows the simulation models and material properties of the molding materials. The LSR injection mold involved conformal heating and cooling channels. The number of meshes for the optical convex lens, LSR injection mold, filling system, and two channels were 2216; 1,344,551; 121; 5800, and 477,176, respectively. A three-dimensional, cyclic, transient heat transfer problem with convective boundary conditions on both the mold and mold base surfaces was set in the simulation. The material property information of the molding materials included a viscosity chart and pressure–volume–temperature diagram. It is well-known that viscosity depends on temperature, shear rate, and pressure because it is the index of material flow resistance. Viscosity will rise sharply when a material reaches a certain temperature at a specific heating rate. As the temperature rises, the volume of the material will begin to expand. The greater the pressure on the material, the smaller its volume as the temperature rises. The seed size for the product, conformal heating channel, and conformal cooling channel was 0.1 mm. The seed size for the mold base was 1 mm. The boundary layer mesh had 5 layers.

**Figure 4:**
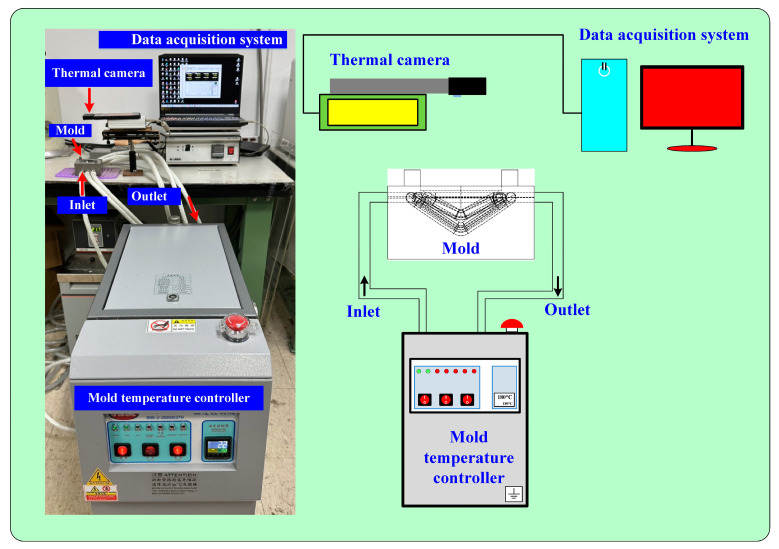
Measuring of the surface temperature of an LSR injection mold using a thermal camera.

**Figure 5:**
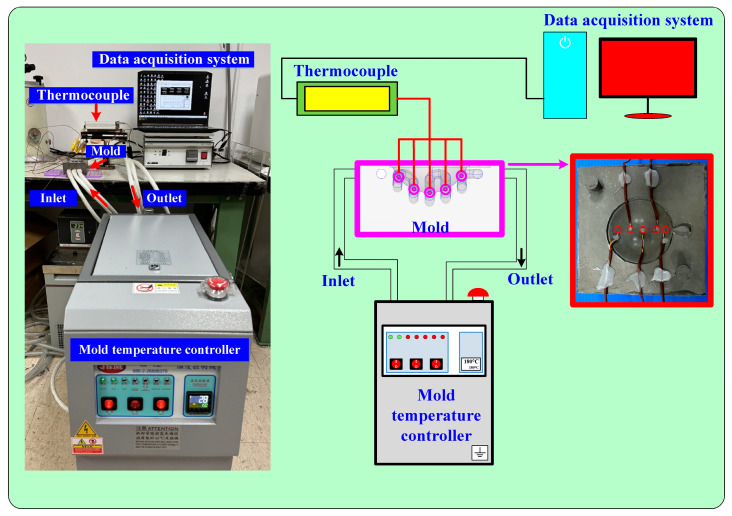
Measuring of the surface temperature of an LSR injection mold using five thermocouples.

To investigate temperature uniformity in the heated cavity, night heating oil temperatures (60, 80, 100, 120, 140, 150, 160, 170, and 180 °C) were employed. Figure 4 shows the measurement of the surface temperature of an LSR injection mold using a thermal camera. Figure 5 shows the measurement using five thermocouples. The experiment set-up for measuring mold surface temperature included an infrared thermal imager (BI-TM-F01P, Panrico trading Inc., New Taipei City, Taiwan), five k-type thermocouples (C071009-079, Cheng Tay Inc., Taipei, Taiwan) with a measurement sensitivity of ±1 °C, a heating oil temperature controller (JSO—1020E, Jie-Seng Inc., New Taipei, Taiwan), and a data acquisition system (MRD-8002L, IDEA System Inc., New Taipei City, Taiwan). The value of center-line average surface roughness (Ra) was used to measure the surface roughness of the LSR injection mold. The measuring range was 250 × 250 µm and a white-light interferometer (7502, Chroma Inc., New Taipei City, Taiwan) was used.

## 3. Results and Discussion

**Figure 6:**
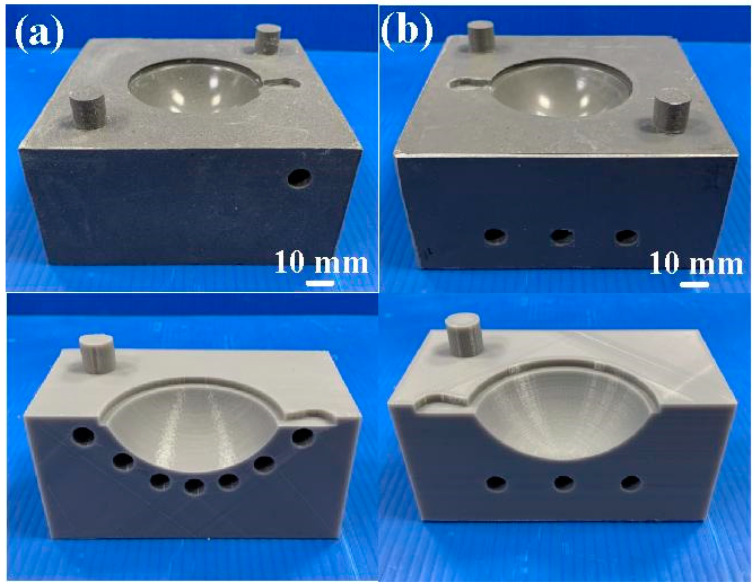
LSR injection mold with (**a**) conformal and (**b**) conventional heating channel.

Figure 6 shows the LSR injection mold with conformal and conventional heating channels. The mold surface temperature was numerically investigated by the Moldex3D molding simulation software using five sensor nodes on the mold surface.

**Figure 7:**
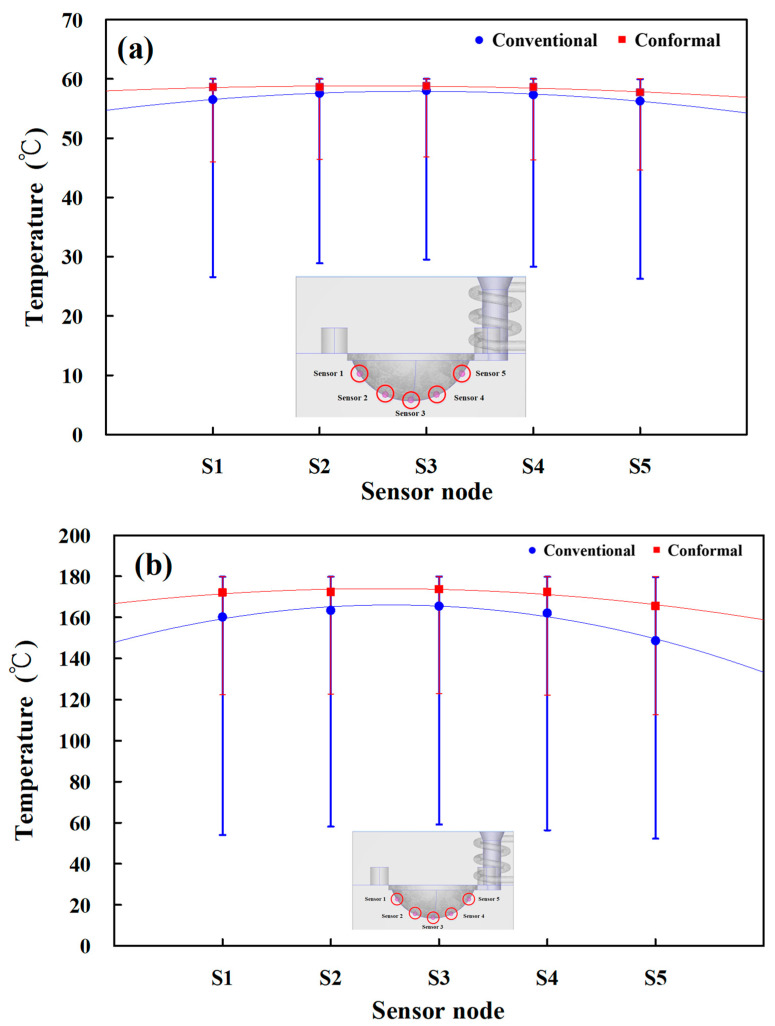
Numerical simulation results of temperature distributions of the five sensor nodes on the mold surface for two different LSR injection molds with the heating oil temperature of (**a**) 60 and (**b**) 180 °C.

**Figure 8:**
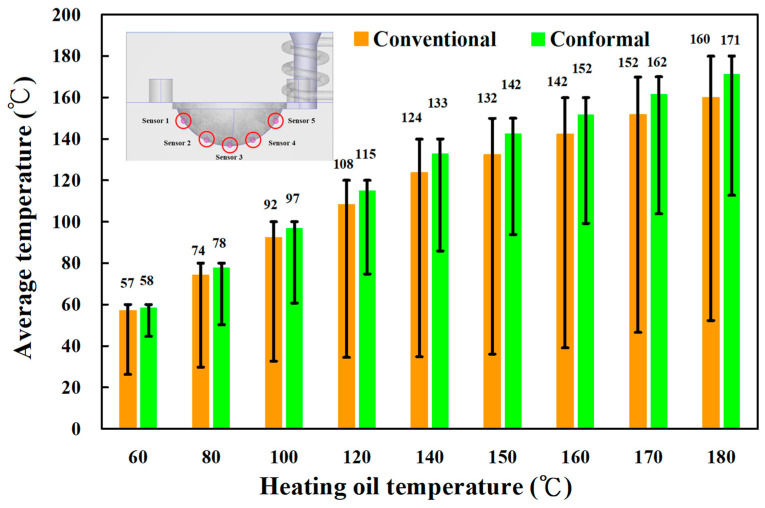
Numerical simulation results of average surface temperature for an LSR injection mold with two different heating systems.

**Figure 9:**
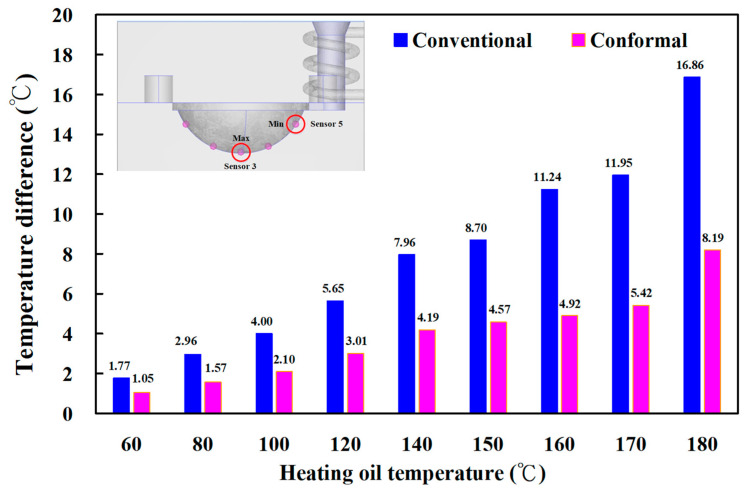
Temperature difference for sensor nodes 3 and 5 for two different LSR injection molds with a heating oil temperature of 60, 80, 100, 120, 140, 150, 160, 170, and 180 °C.

Figure 7 shows the numerical simulation results of temperature distributions of the five sensor nodes on the mold surface for two different LSR injection molds with a heating oil temperature of 60, 80, 100, 120, 140, 150, 160, 170, and 180 °C. The results showed that the average temperature of sensor node 3 was the highest because it was closer to the heating system. However, the average temperature of sensor node 5 was the lowest because it was closer to the conformal cooling system. Figure 8 shows the numerical simulation results of the average temperature of the mold surface for an LSR injection mold with two different heating systems. The upper limit is the highest temperature of the mold surface, and the lower limit is the lowest temperature of the mold surface. For the LSR injection mold with the conventional heating channel, the average surface temperature was 57.2, 74.2, 92.2, 108.3, 123.7, 132.3, 142.3, 151.6 and 160 °C, respectively. It should be noted that the average temperature of the mold surface was up to 58.5, 77.7, 96.8, 115.0, 133.0, 142.5, 151.9, 161.7, and 171.3 °C, respectively. Based on the above data, two results were found in this study. One is that the uniformity of the mold surface for the LSR injection mold with the conformal heating channel was better than for the mold with the conventional heating channel. This meant that the temperature difference between the highest and lowest surface temperatures for the mold with the conformal heating channel was small. Figure 9 shows the temperature difference for sensor nodes 3 and 5 for two different LSR injection molds with different heating oil temperatures. The temperature difference for the mold with the conventional heating channel was about 1.77, 2.96, 4.00, 5.65, 7.96, 8.70, 11.24, 11.95, and 16.86 °C, respectively. However, for the mold with the conformal heating channel, the difference was about 1.05, 1.57, 2.10, 3.01, 4.19, 4.57, 4.92, 5.42, and 8.19 °C, respectively. The other is that the heating rate of the mold surface for LSR injection mold with conformal heating channel was faster.

**Figure 10:**
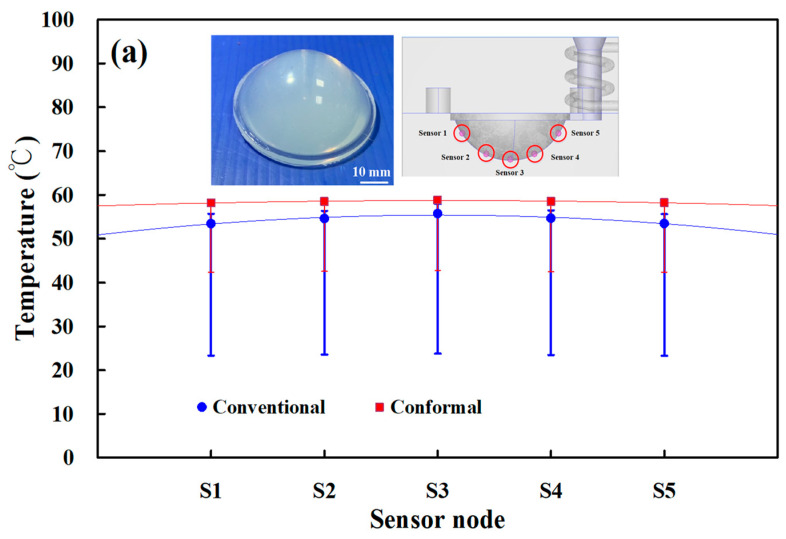

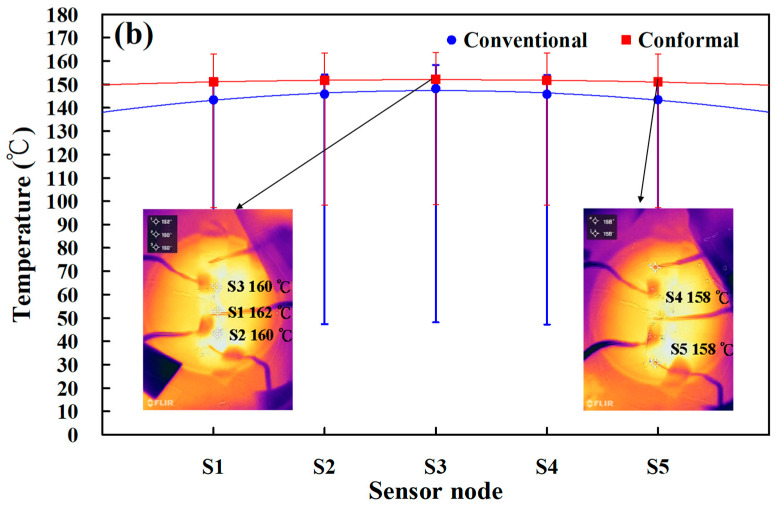
Experimental results of temperature distributions of the five sensor nodes on the mold surface for two different LSR injection molds with the heating oil temperature of (**a**) 60 and (**b**) 180 °C using a thermal camera.

**Figure 11:**
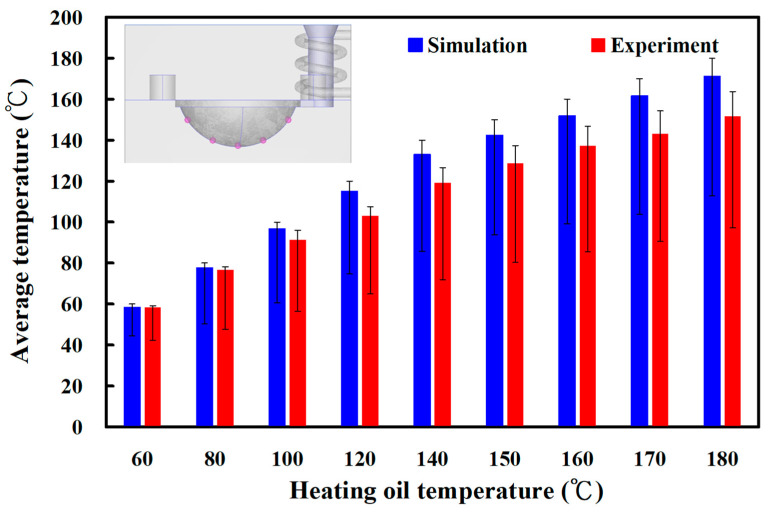
Experimental and simulation results of average temperature of the mold surface for an LSR injection mold with conformal heating.

**Figure 12:**
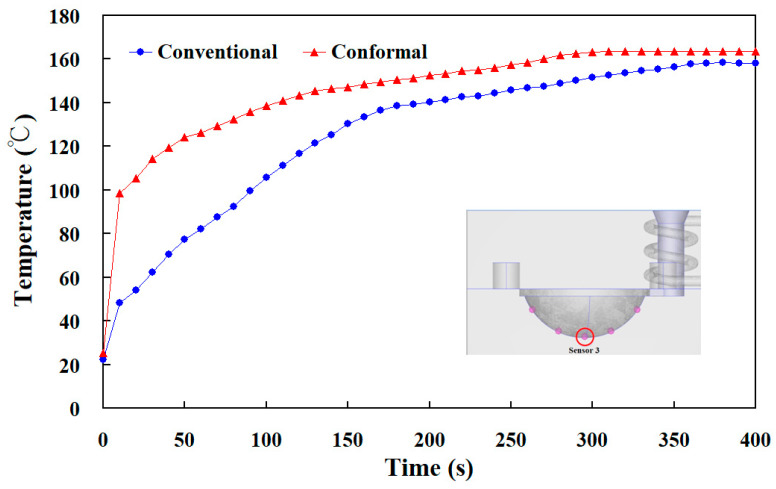
Experimental results of the temperature history of sensor node 3 for two different LSR injection molds with a heating oil temperature of 180 °C using a thermocouple.

**Figure 13:**
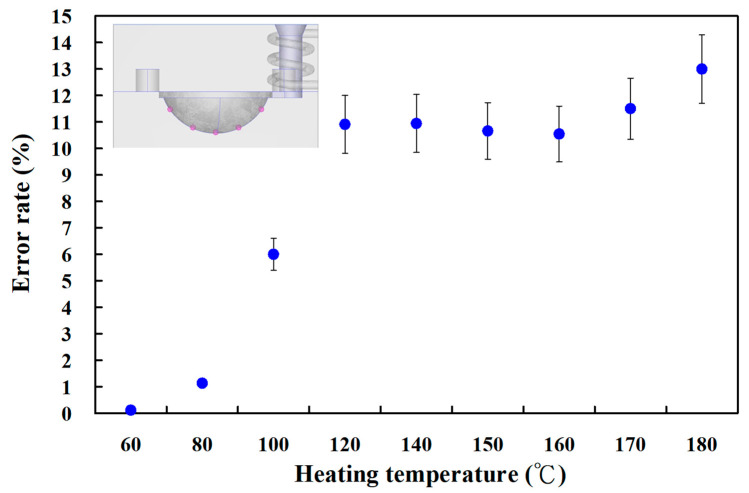
Error rate between experimental and simulation results of average temperature of the mold surface for LSR injection mold with conformal heating.

Figure 10 shows the experimental temperature distribution results of the five sensor nodes on the mold surface for two different LSR injection molds with a heating oil temperature of 60 and 180 °C using a thermal camera. The experimental results were consistent with the simulation results, showing the uniformity of the mold surface for an LSR injection mold with a conformal heating channel was better than one with a conventional heating channel. Figure 11 shows the experimental and simulation results of the average surface temperature for an LSR injection mold with conformal heating. It should be pointed out that the average temperature of the mold surface (y) was estimated by the heating oil temperature (x) according to the prediction equation of y = −0.7999x^2^ + 19.477x + 40.13 with a correlation coefficient of 0.9984. The trend of the average temperature of the five sensing nodes on the mold surface from the experimental results was consistent with the simulation results. However, the average temperature of the experiment was lower than the that of the simulation. For example, the maximum mold surface temperature for an LSR injection mold with conformal heating channel reached only 150 °C when a heating oil temperature of 180 °C was used to vulcanize the optical convex lens. The possible reason is the different environmental conditions between the experiment and simulation. In practice, an oil pipe was exposed to the atmosphere causing a heat transfer loss in the process of heating the oil. This phenomenon was confirmed by the temperature history of sensor node 3 obtained by a thermocouple. Figure 12 shows the experimental results of the temperature history of sensor node 3 for the two LSR injection molds with a heating oil temperature of 180 °C using a thermocouple. Figure 13 shows the error rate between the experimental and simulation results of the average temperature of the mold surface for the LSR injection mold with conformal heating. Based on the experimental results, the simulation error rate was about 0.110, 1.130, 6.00, 10.910, 10.940, 10.660, 10.540, 11.50, and 13.00%, respectively. The error rate of the simulation results was about 8.31% based on the experimental results.

**Figure 14:**
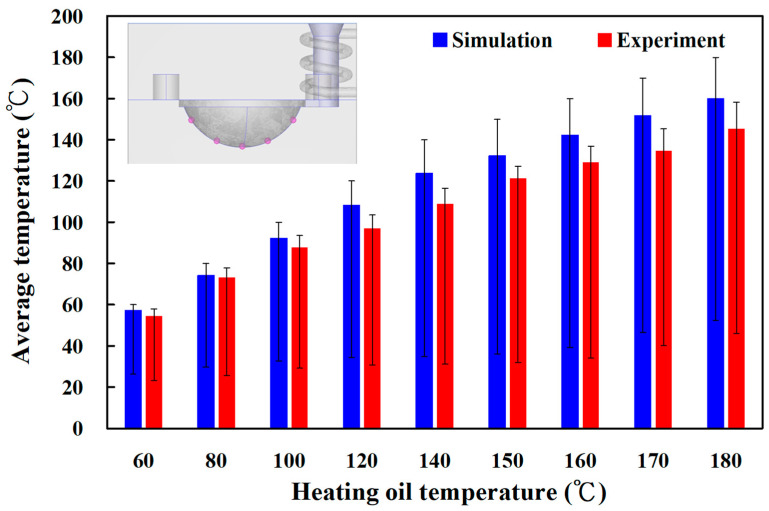
Experimental and simulation results of the average temperature of the mold surface for an LSR injection mold with conventional heating.

**Figure 15:**
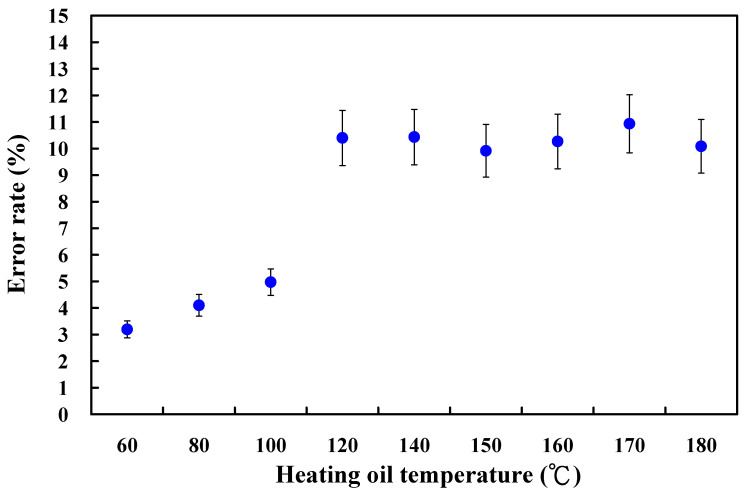
Error rate between the experimental and simulation results of the average mold surface temperature of an LSR injection mold with conventional heating.

**Figure 16:**
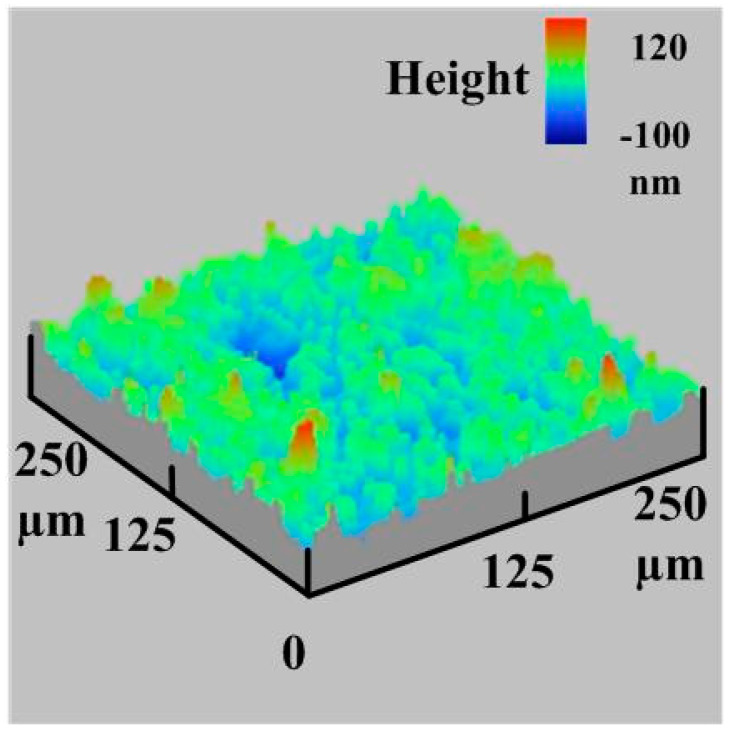
Surface analysis of the LSR injection mod.

The same phenomenon was found in the LSR injection mold with conventional heating. Figure 14 shows the experimental and simulation average surface temperature results. Figure 15 shows the error rate between the experimental and simulation results. Based on the experimental results, the simulation error rate was about 3.20, 4.10, 4.97, 10.40, 10.43, 9.91, 10.27, 10.93, and 10.08%, respectively. Figure 16 shows the surface analysis of the LSR injection mold. The average surface roughness the LSR injection mold was about 174 nm.

This paper presented a conformal heating channel to enhance the temperature uniformity of the mold surface in the LSR injection molding of an optical convex lens. It should be noted that the fabricated LSR injection mold fulfilled sustainable development goals [23,24,25,26,27]. The above-described findings provided the greatest application potential in the design stage of a new optical convex lens. In this study, two different sets of rapid tools were implemented using a mixture comprising of Al powder and epoxy resin. Unfortunately, the mechanical property [28] of the rapid tool [29] was inferior to conventional mold steel [30]. Thus, improving the mechanical properties of the fabricated LSR injection mold [31,32,33] by adding stainless steel or iron powder [34] is an interesting research topic. In addition, it was interesting to observe the surface roughness [35] of the 3D print mold on the surface of the lens using a scanning electron microscope [36] or a 3D optical profilometer [37]. Also, it was interesting to analyze the chemical composition and changes that produced variation in fabrication temperature using Raman spectroscopy [38] or attenuated total reflectance using Fourier-transform infrared spectroscopy [39]. This research is ongoing and the results will be presented in a later work.

## 4. Conclusions

Traditionally, the use of plastic lenses has distinct drawbacks: they have short service life and are easily scratched. An optical-grade LSR lens can provide safety and aging resistance. The major objective of this study was to enhance the temperature uniformity of the mold surface in the injection molding of LSR using the conformal heating channel. The main conclusions from the experimental work in this study are as follows:

1. The uniformity of the mold surface for LSR injection mold with the conformal heating channel was better than for the mold with the conventional heating channel.
2. The experimental results showed that the average temperature of the mold surface (y) could be predicted by the heating oil temperature (x) according to the prediction equation of y = −0.7999x^2^ + 19.477x + 40.13 with a correlation coefficient of 0.9984.
3. The experimental results showed that the trend of the average temperature of five sensor modes was consistent with the simulation results.

The error rate of the simulation results was about 8.31% based on the experimental result of LSR injection mold with the conformal heating channel.

## Data Availability

Data and materials are available.
